# A Nux Case, “Model Cure”

**Published:** 1894-04

**Authors:** G. J. Waggoner

**Affiliations:** Larned, Kansas


### A NUX CASE. "MODEL CURE."

G. J. Waggoner, M. D., Larned, Kansas.

On Friday evening, January 13th, 1894, was called to see Mrs. Ida D., æt. twenty, in puerperal month. For several days had pain in stomach and bowels, and most of all, in rectum and anus, with "piles," and constant urging to stool with little or no result, rectum protruding with sense of light closure. Had taken freely of Wright's vegetable (?) pills, which aggravated all the symptoms without moving bowels. Felt cross, vindictive, malicious. Gave a dose of Nux.$^{1m}$, dry on tongue, and powder of Sac-alb. of which to take a dose once in two hours till relieved. Wanted to know what good that little sugar was going to do in a case like hers? Was told to wait and see; that she would be all right in the morning. Report next morning was that all the trouble ceased in a few minutes after taking first dose; slept well all night, and had natural easy stool in the morning.

The family are now willing to take "sugar," or anything else instead of "medicine."

